# Management of acute renal colic in the UK: a questionnaire survey

**DOI:** 10.1186/1471-227X-4-5

**Published:** 2004-12-07

**Authors:** Tunji A Lasoye, Philip M Sedgwick, Nilay Patel, Chas Skinner, Nadeem Nayeem

**Affiliations:** 1 Accident and Emergency Department, King's College Hospital, London, England, UK; 2 Department of Community Health Sciences, St George's Hospital Medical School, London, England, UK; 3 Department of Urology, Churchill Hospital, Oxford, England, UK; 4 Department of Psychology, University of Southampton, Southampton, England, UK; 5 Accident and Emergency Department, University Hospital Lewisham, London, England, UK

## Abstract

**Background:**

There is great variation in the Accident and Emergency workload and location of Urology services in UK hospitals. This study investigated the relationship of the initial management of acute renal colic with the department workload plus local facilities including location of X-ray and urology services in UK Accident and Emergency (A&E) departments.

**Methods:**

A&E departments in each of the 11 UK Deanery regions were stratified based on departmental workload, namely <30,000 (*small*); 30,000 to 50,000 (*medium*); 50,000 to 80,000 (*large*) and >80,000 (*very large*) patients per year. One third of departments were selected in each group leading to a sample size of 106. A questionnaire was administered. Associations between categorical variables were investigated using the chi-squared test and when not valid, Fisher's Exact test was employed. Differences between groups in ordinal variables were investigated using the Mann-Whitney test.

**Results:**

All questionnaires were returned. Twenty-nine units (27.4%) did not perform any radiological investigation on renal colic patients. The number of radiological investigations that were available to departments was associated with workload (*P *= 0.003); with 57.1% of the *small *departments performing none and at least 82.8% of units in the other categories performing at least one. Of those departments with X-ray facilities in or adjacent to the department, 63% performed an intravenous urography (IVU) compared to 25% of those departments without (*P *= 0.026). Of those departments with on-site urology services, 86% performed at least one radiological investigation compared to 52% of units without such services (*P *= 0.001). Department workload was associated with the first choice analgesia (NSAIDs or parenteral opiates) (*P *= 0.011). Of the *small *departments, 64.3% used NSAIDs, 21.4% used parenteral opiates and 14.3% used neither. In comparison, NSAIDS were used by at least 87%, and opiates by at most 12.5% of units in each of the other three categories of department workload.

**Conclusions:**

Over a quarter of UK A&E departments did not perform any radiological investigations and some departments do not even offer renal colic patients any analgesia. Patient management was associated with departmental workload, location of X-ray and Urology services. National guidelines are needed to ensure optimum care for all patients.

## Background

Upon presentation to the A&E department, suspected acute renal colic patients must have a clinical examination and radiological investigations to confirm the diagnosis. Without radiological investigations, life-threatening conditions such as abdominal aortic aneurysm and ectopic pregnancy may be misdiagnosed as renal colic. However, a delay in the diagnosis is possible as the facilities needed for the diagnosis are sometimes not based in the same hospital as the A&E department. In the UK, great variation exists between Accident and Emergency services in their workload as measured by the number of new patients seen per year[1]. Larger A&E departments tend to be located in those teaching hospitals that have most specialist services on-site, therefore facilitating adequate investigation of suspected renal colic. Every A&E department in the UK should be able to perform initial assessment and investigation of suspected renal colic, provide pain relief and refer appropriately, irrespective of the location of urology services. If acute renal colic presented to primary care then the patient would be rapidly referred to secondary care, namely an A&E department. The aims of this study were to investigate the initial management of acute renal colic in UK A&E departments and if practice was related to department workload, plus the location of X-ray and urological services in relation to the A&E department.

## Methods

The handbook of the British Association for Accident and Emergency lists a total of 311 A&E departments in the UK[1]. The UK is divided into 11 so-called Deanary regions that represent geographical areas. These units were categorised according to their workload (number of new patients seen per year) as follows: *Small*- less than 30,000, *Medium*- 30,000 to 50,000, *Large *-50,000 to 80,000 and *Very Large*-more than 80,000. For each of the four-workload categories in each of the 11 Deanery regions, every third unit was selected resulting in a total of 106 (34.1%) departments.

A questionnaire was administered by post to the 106 departments (see Appendix 1) requesting details about the location of X-ray, location of Urology services plus current practice in the investigation and management of pain in acute renal colic patients. A covering letter was included indicating that the purpose of the survey was to collect information about practice when patients present to the A&E department and not their subsequent management. The most senior medical member of each department was invited to complete the questionnaire. Over a period of nine months, each of the 106 departments returned a completed questionnaire. A total of 35 departments did not respond initially and they were sent a second questionnaire by post as a reminder. Ten departments did not respond to the second questionnaire and these were followed up with a telephone call. Consultants completed the questionnaire in 74.5% (n = 79) of units. Middle grade doctors who had been in post for at least six months completed the remaining 27 (25.5%) questionnaires.

### Statistical methods

The Chi-squared test (test statistic denoted by χ^2^) was used to investigate the following associations: a) the location of X-ray and intravenous urography; b) location of Urology services and total number of investigations performed and c) categorised departmental workload with the investigations performed and also the analgesics used. When the Chi-squared test was invalid, Fisher's Exact test (test statistic denoted by FI) was employed. The Chi-Squared was considered to be invalid if more than 20% of the cells had an expected value less than five or if one of the cells had an expected value less than one [2]. The categorised departmental workload groups were compared in the number of films used during an IVU procedure using the Kruskal-Wallis test (test statistic approximated to the Chi-Squared distribution and denoted by χ^2^) [3]. Degrees of freedom were abbreviated to df. The critical significance level was 0.05. All statistical analyses were performed using SPSS for Windows (version 11).

## Results

### On-site services

Of the 106 departments, a total of 94 (88.7%) had X-ray facilities located in the department. A greater proportion of those departments that have X-ray facilities within their premises used the Intra-Venous Urogram (IVU) option compared to those departments without these facilities [n = 59 (62.5%) versus n = 3 (25%); FI = 6.03, df = 1, *P *= 0.026].

Urology was located within the hospital for 64 (60.4%) departments. The total number of radiological investigations [IVU, Ultrasound Scan (USS) or Computed Tomogram (CT)] that were available to units was categorised as none, one and two or more. Those departments that had urology on-site had more radiological options available than those without (*P *= 0.001) (see Table 1). At least one radiological option was used by 85.9% (n = 55) of those units with on-site urology services compared to 52.3% (n = 22) of units without.

**Table 1:** Association between total number of radiological investigations performed and location of urology services.

	**Total Number of Investigations**			
**Location of Urology services**	**None**	**One**	**Two or three**	**Total**
**Within Hospital**	9 (14.1%)	36 (56.3%)	19 (29.7%)	64 (60.4%)
**Outside Hospital**	20 (47.6%)	13 (31.0%)	9 (21.4%)	42 (39.6%)

Percentages in brackets are those within the category of the location of urology services; those in the 'total' column are those for the whole sample (n = 106). There was a significant difference between hospitals as regards their location of services in the number of investigations performed (χ^2 ^= 14.6, df = 2, *P *= 0.0007).

None of the departments in our study routinely used nuclear medicine to investigate renal colic.

### Radiological investigations

#### Intra-Venous Urogram (IVU)

A significant relationship existed between department workload and if an IVU option was available (*P *= 0.001) (see Table 2). An IVU option was available to 28.6% of the *small *departments, compared to at least 62.5% of those units in the larger categories, namely the *medium*, *large *and *very large *departments.

**Table 2:** Tabulation of department workload by radiological investigations performed plus total number of investigations, and number of films used in IVU investigations.

	**Number of new patients per year**					
			
	**< 30,000**	**30,000 to 50,000**	**50,000 to 80,000**	**>80,000**	**All departments**	**Test statistics**
**IVU performed**						
No	20(71.4%)	8 (22.9%)	13 (37.1%)	3 (37.5%)	44 (41.5%)	FI = 15.54, df = 3, *P *= 0.001
Yes	8 (28.6%)	27 (77.1%)	22 (62.9%)	5 (62.5%)	62 (58.5%)	
**USS performed**						
No	21 (75.0%)	25 (71.4%)	17 (48.6%)	4 (50.0%)	67 (63.2%)	χ^2 ^= 6.52, df = 3, *P *= 0.089
Yes	7 (25.0%)	10 (28.6%)	18 (51.4%)	4 (50.0%)	39 (36.8%)	
**CT performed**						
No	27 (96.4%)	33 (94.3%)	32 (91.4%)	5 (62.5%)	97 (91.5%)	FI = 6.87, df = 3, *P *= 0.056
Yes	1 (3.6%)	2 (5.7%)	3 (8.6%)	3 (37.5%)	9 (8.5%)	
**Total number of investigations**						
None	16 (57.1%)	6 (17.1%)	6 (17.1%)	1 (12.5%)	29 (27.4%)	FI = 18.85, df = 6, *P *= 0.003
One	9 (32.2%)	21 (60.0%)	16 (45.7%)	3 (37.5%)	49 (46.2%)	
Two or three	3 (10.7%)	8 (22.9%)	13 (37.2%)	4 (50.0%)	28 (26.4%)	
**If IVU, total Number of films**						
n	8	27	22	5	62	χ^2 ^= 6.68, df = 3, *P *= 0.083
mean	3.0	2.9	2.6	1.6	2.7	
standard deviation	1.14	0.97	1.05	0.89	1.10	
median	3	3	3	1	3	
lower quartile	2.0	3.0	2.0	1.0	2	
upper quartile	4.5	3.0	3.0	2.5	3	

Percentages in brackets are those of the grouped department workload; those in the "All departments" column are of the 106 units. The test statistics comparing the four groups of department size are displayed.

The relationship between department workload and the average number of films used when an IVU was performed is shown in Table 2. Of the 106 departments, 43 (40.6%) did not undertake an IVU leaving a total of 63 (59.4%) units for analysis. All of these 63 departments used between one and five films per IVU investigation except for the *very large *category where the greatest number of films used by a department was three. The *very large *departments used a median number of a single film whilst the other three categories of department size used a median number of three films. Although there was a tendency for fewer films to be used as departmental size increased, this just failed to reach statistical significance at the 5% level (*P *= 0.083).

#### Ultrasound Scan (USS)

There was no statistically significant relationship between department workload and if an USS option was available (*P *= 0.089). However, at least half of the *large *and the *very large *units used USS compared to less than 30% of the departments in the *small *and *medium *sized categories (see Table 2). Overall, 36.8% (n = 39) of departments were able to perform an USS.

#### Computerised Tomogram (CT) Scan-Helical CT

The relationship between department workload and if a CT scan was available just missed statistical significance (*P *= 0.056) (see Table 2). Of the *very large *departments, 37.5% (n = 3) could perform a CT scan compared to less than 10% of the units in each of the *small*, *medium *and *large *categories.

#### Total number of radiological investigations

A total of 29 units (27.4%) did not perform any radiological investigations. The relationship between the total number of investigations available and department workload was statistically significant (*P *= 0.003) (see Table 2). No radiological investigations were carried out by 16 (57.1%) of the *small *departments whilst at least 83% of the units in each of the other three departmental workload categories were able to perform at least one radiological investigation. Exactly half (n = 4) of the *very large *departments had at least two options available.

### Choice of analgesia

There was a statistically significant relationship between department workload and the first choice analgesia: either NSAIDs (Diclofenac or Ketorolac) or parenteral opiates (*P *= 0.011) (see Table 3). Parenteral opiates were used by 21.4% (n = 6) of the *small *departments compared to at most 12.5% of units in the other workload categories. Neither NSAIDs nor parenteral opiate was used by four (14.3%) of the *small *departments and one *large *department; one of these *small *units plus the *large *department reported using codydramol (a combination of paracetamol with dihydrocodeine). Of the 106 departments, 91 (85.8%) used NSAIDs including 86 (81.1%) – diclofenac and five (4.7%)- ketorolac as the first choice analgesia. Of the 86 departments that used diclofenac, 68 (79.1%) routinely used the intra-muscular route, 17 (19.7%) the rectal route and one (1.2%) administered it orally.

**Table 3:** First choice analgesic (either NSAIDs, Parenteral opiates or neither) by department workload (n = 106).

**First choice analgesia (NSAIDs or Parenteral opiates)**	**Number of new patients per year**				**All departments**
		
	**< 30,000**	**30,000 to 50,000**	**50,000 to 80,000**	**> 80,000**	
**None used**	4 (14.3%)	0	1 (2.9%)	0	5 (4.7%)
**NSAIDs**	18 (64.3%)	34 (97.1%)	32 (91.4%)	7 (87.5%)	91 (85.8%)
**Parenteral opiates**	6 (21.4%)	1 (2.9%)	2 (5.7%)	1 (12.5%)	10 (9.4%)

Percentages in brackets are those of the grouped departmental workload; those in the "All departments" column are of the 106 units. There was significant difference between department workloads in first choice analgesia (FI = 13.49, df = 6, *P *= 0.011).

## Discussion

This study reports the initial management of renal colic irrespective of which specialty team carried out the management. Traditionally, renal colic was confirmed by IVU alone [4] although the use of USS and helical CT scans has increased in current practice [5]. A study of a single department reported that up to 37% of patients with suspected renal colic were investigated with ultrasound, although this included mainly patients with allergy to the contrast used in IVU and those in early pregnancy when irradiation needs to be avoided [6]. There may to be an upward trend in the use of USS in A&E departments due to the current drive for USS by non-radiologists [7-9]. Our study found that only a quarter of UK units used USS although these included at least half of each of the *large *and *very large *departments.

Radiological investigations confirm or refute a diagnosis of renal colic. If the diagnosis is refuted, then the clinician is prompted to consider other diagnoses. We found that over a quarter of departments (27.4%; n = 29) did not perform any radiological investigation (see Table 2). This is of concern since it has been reported that renal colic is one of the most common misdiagnoses in catastrophic abdominal conditions including ectopic pregnancy and abdominal aortic aneurysm [10]. The concern is greatest for those departments in the *small *category; nearly 60% of them did not routinely perform any radiological investigations and they may be located in remote areas lacking specialist surgical facilities such as on-site vascular surgery. When departments are isolated with minimal specialist back up, an early diagnosis is crucial, so that patients with other abdominal conditions requiring urgent specialist management can be appropriately referred. An IVU can be easily done in the X-ray department; a negative IVU should prompt the clinician to consider an alternative diagnosis to renal colic and this, in our opinion should not be beyond the reach of any A&E department in the UK.

Intra-venous urography was performed by a significantly greater proportion of those departments with X-ray facilities within the unit compared to those with X-ray facilities located elsewhere. This would suggest that if all A&E departments had X-ray facilities located within the unit, the potential for misdiagnosis would be minimised since all units would then be more likely to perform at least an IVU.

Those hospitals that had on-site urology services performed more investigations than those sites without (see Table 1). In particular nearly half of those hospitals with urology services located outside the hospital did not perform any investigations at all compared to 14% of those hospitals with on-site services. This potentially means that patients with conditions other than renal colic are sent to a urology clinic with the consequence that their management is delayed.

We found that when an IVU was performed, the larger units used fewer films although this relationship just missed statistical significance (see Table 2). This finding suggests that adequate information to diagnose renal colic might be obtained by using only one film, in keeping with previous findings [11]. However, these suggestions need to be verified by further research.

We found that less than 10% of UK A&E departments use a CT scan in the assessment of renal colic and the association with departmental workload just missed statistical significance at the 5% level (see Table 2). A CT scan was available to 37.5% of the *very large *departments compared to less than 10% in the other sized categories. The main difficulty with performing a CT scan in an A&E setting is that interpretation of the images requires urologists or radiologists who are not always available [5]. When appropriate personnel are available, CT should be the favoured investigation as it has been shown not only to diagnose urinary tract calculi accurately but also provide other diagnoses. The choice of investigation in some of the units that reported using more than one type of radiological investigation may have been influenced by availability of the required personnel, as both USS and CT require some expertise. However, this study did not investigate this aspect of practice.

Previous research has shown the efficacy of NSAIDS in renal colic [12-17]. We found that 85.8% of UK A&E departments use NSAIDS. Intra-venous ketorolac reported to have the fastest onset of action and equal analgesic properties to other NSAIDS, was used by only 4.7% of units although its use may have been precluded by difficulty with venous access. Intra-muscular diclofenac was routinely used in 64% of departments despite the problems associated with this route including discomfort at the injection site and the potential for sterile abscess formation [16]. Whilst the rectal route is favoured over the intra-muscular route since it is equally effective and avoids possible injection site problems, only 16% of all departments in this study reported using it.

In spite of the proven efficacy of NSAIDS, we found that nearly 10% of all A&E departments used parenteral opiates as the analgesic of first choice. Given that opiate administration requires checking and crosschecking by at least two nurses, there will inevitably be a delay in relieving the patients' pain. Parenteral opiates would be better as second-choice analgesic in our opinion.

We found five departments, including four in the *small *and one in the *large *workload categories that did not use either NSAIDs or parenteral opiates in suspected renal colic. The *large *and one of the *small *departments prescribed Codydramol. The other three *small *departments referred renal colic patients directly to a urology team off-site without even offering analgesia. Why these departments adopted this approach was unclear. Nonetheless, these findings were of concern since an A&E department would be expected to at least consider offering analgesia to patients that present to them in pain irrespective of the patients' final destination.

There is no reason to suspect that the departments in this study were not representative of A&E units in the UK, since the sample was derived from each of the workload categories in the 11 UK Deanery regions. However, as with any questionnaire study it is difficult to assess the reliability of the answers provided. The most senior individual in the department was invited to complete the questionnaire. However, it is not possible to quantify the bias, if any, that may be introduced by the variation in grade of the respondents. Since nearly three quarters of the questionnaires were completed by consultants one might expect that that the results were reliable. However, it is possible that consultants may not be fully aware of routine practice and therefore the information provided could be inaccurate. Furthermore, as soon as a unit was asked about its current practice through the questionnaire, it may subsequently have adjusted it, particularly if it was sub-optimal. Therefore, the information provided on the questionnaire may reflect the altered, rather than original practice.

## Conclusions

The management of acute renal colic differs between A&E departments in the UK. Local factors may contribute to these differences. The total number of radiological procedures that were available to a unit was positively associated with departmental workload. Of great concern was that a significant proportion of departments overall (27.4%) did not perform any radiological investigation. The concern is greatest for those departments in the *small *category with nearly 60% performing no radiological investigation. Location of X-ray facilities within the A&E premises is associated with whether an IVU is ever performed. Departments with on-site urology have a greater range of radiological investigations to choose from. Furthermore, the *very large *units tended to routinely use fewer films per IVU, with a median number of one compared to three in all other smaller units.

The first choice analgesic used by most units is NSAIDS in keeping with the literature; more departments, however, need to adopt the use of the rectal route for diclofenac in order to avoid the potential complication of the intra-muscular route. The low percentage of departments using parenteral opiates as first-choice analgesic is encouraging as parenteral opiates are better used as second choice in view of the unavoidable delay that occurs before their administration.

The practice in over a quarter of A&E departments in the UK is below standard. There is significant association with departmental workload and location of services such as radiology and Urology relative to A&E. We suggest that national guidelines be developed for the management of acute renal colic in A&E departments to ensure optimum care for all patients. Subsequent to the implementation of any guidelines, we suggest that UK practice is regularly reviewed.

## Competing interests

The author(s) declare that they have no competing interests.

## Authors' contributions

TAL undertook the literature search, designed the questionnaire, participated in the collection and analysis of data, and wrote the paper. PMS undertook the statistical analysis and contributed to the writing of the paper. NN initiated the research, participated in data collection and contributed to the paper. CS performed the initial stratification and selection of units for the study. NP participated in data collection.

## Appendix 1

### Questionnaire on renal colic

1. Name of hospital: ____________________________________________

2. Where is X-ray located? Within or adjacent to A&E □

Distant □

3. Where are urology services located? Same Site as A&E □

Separate Site from A&E □

4. Are the following investigations performed on suspected cases of renal colic?

a) Urinalysis No □ Yes □

b) IVU No □ Yes □

If IVU is performed, please indicate how many films are used □

c) CT No □ Yes □

d) USS No □ Yes □

e) Nuclear Medicine No □ Yes □

5. Which of the following analgesics are given on presentation?

a) Codydramol Other Oral No □ Yes □

b) NSAIDS No □ Yes □

If NSAIDS used; which one: (indicate route below)

i. Intra-muscular □

ii. Oral □

iii. Rectal □

iv. Intra-venous □

c) Parenteral opiate No □ Yes □

If parenteral opiates are used then please indicate if first or second choice:

i. First choice □

ii. Second choice □

## Pre-publication history

The pre-publication history for this paper can be accessed here:


